# Identification of key genes and immune cell infiltration in recurrent implantation failure: A study based on integrated analysis of multiple microarray studies

**DOI:** 10.1111/aji.13607

**Published:** 2022-08-15

**Authors:** Xue Feng, Xiaolin Meng, Shuaiqingying Guo, Kezhen Li, Lingjuan Wang, Jihui Ai

**Affiliations:** ^1^ Reproductive Medicine Center Tongji Hospital Tongji Medical College Huazhong University of Science and Technology Wuhan Hubei China; ^2^ Department of Gynecology and Obstetrics Tongji Hospital Tongji Medical College Huazhong University of Science and Technology Wuhan Hubei China

**Keywords:** hub genes, immune cell infiltration, recurrent implantation failure

## Abstract

**Problem:**

Recurrent implantation failure (RIF) refers to a challenging topic in assisted reproductive technology (ART), the etiology of which may be attributed to impaired endometrial receptivity; however, the precise pathogenesis of RIF has not been thoroughly elucidated.

**Method of study:**

Four RIF microarray datasets were obtained from the Gene Expression Omnibus database and integrated by the “sva” R package. The differentially expressed genes (DEGs) were analyzed using the “limma” package and then GO, KEGG, GSEA, and GSVA were applied to perform functional and pathway enrichment analysis. The immune cell infiltration in the RIF process was evaluated by the CIBERSORT algorithm. Finally, the hub genes were identified through the CytoHubba and subsequently verified using two items of external endometrial data.

**Results:**

236 genes were differentially expressed in the endometrium of the RIF group. Functional enrichment analysis demonstrated that the biological functions of DEGs were mainly correlated to the immune‐related pathways, including immune response, TNF signaling pathway, complement and coagulation cascades. Among the immune cells, γδ T cells decreased significantly in the endometrium of RIF patients. In addition, the key DEGs such as *PTGS2*, *FGB*, *MUC1*, *SST*, *VCAM1*, *MMP7*, *ERBB4*, *FOLR1*, and *C3* were screened and identified as the hub genes involved in the pathogenesis of RIF.

**Conclusions:**

Abnormal immune response regulation of endometrium contributes to the occurrence of RIF, and γδ T cells may be the pivotal immune cells causing RIF. At the same time, the novel hub genes identified will provide effective targets for the prediction and therapy of RIF.

## 1 INTRODUCTION

The emergence of assisted reproductive technology (ART) has enabled countless infertile couples to achieve clinical pregnancy, but nearly 10% of patients still fail to conceive successfully due to recurrent implantation failure (RIF).^1^ RIF is usually defined as failure to achieve a clinical pregnancy after no less than three consecutive high‐quality embryo transfers or multiple transfers of at least 10 embryos.^2^ RIF has caused serious mental stress and a huge financial burden to patients. Therefore, it is of great theoretical and clinical value to identify the molecular markers of RIF and clarify its possible mechanism.

The pathogenesis of RIF remains elusive, and poor endometrial receptivity is considered to be the decisive cause of RIF.^3^ Endometrial receptivity is regulated by many factors, and abnormal gene expression is the key factor leading to deficient endometrial receptivity. For example, it has been reported that MUC1,^4^ HOXA10,^5^ and LIF^6^ are abnormally expressed in RIF patients. In recent years, the rapid development of sequencing technology provides a new method for the study of the pathogenesis of RIF. Studies have found that there may be abnormal gene expression profiles in RIF patients’ endometrium, which greatly promotes the understanding of the pathogenesis of RIF.^7^, ^8^, ^9^ However, due to the relatively small sample size and inconsistency caused by different array platforms or bioinformatics methods, the microarray studies using endometrial transcriptome to identify key molecules related to RIF eventually bring about differences between studies and potential deviations in the results.

Implantation is a physiological inflammatory process, which is also a process of destruction and reconstruction while keeping a balance of immune microenvironment between the embryo and maternal endometrium. Local immune dysfunction can reduce endometrial receptivity and consequently lead to RIF. There are many kinds of immune cells in the endometrium, such as natural killer cells (NK cells), macrophages, dendritic cells (DC), T cells, and so on, which are essential in regulating endometrial receptivity and embryo implantation.^10^ For example, Ee Von Woon et al. observed a significant increase in the level of uterine NK cells in RIF, indicating that there may be a potential immune environment disorder in the endometrium of patients with RIF.^11^ The polarization balance between M1 and M2 macrophages will affect the invasion and migration of trophoblast.^12^ Su Liu et al. found that ILT‐4^+^ DC in the peripheral blood and endometrium of RIF patients decreased during the mid‐luteal phase, and it is speculated that ILT4^+^ DC can maintain pregnancy immune tolerance by inducing Foxp3^+^ Treg.^13^ The reproductive immune system is an extremely complex entity since it involves the interaction and regulation of a variety of immune cells and related cytokines, so a single immune cell detection cannot evaluate the whole picture of the immune system. However, at present, the research exploring immune cells in the endometrium is still scattered, and there is a lack of systematic and comprehensive analysis of RIF‐related immune regulation and immune cell infiltration.

In this study, we integrated several RIF microarray studies to identify gene‐specific expression patterns and found that immune response regulation took part in the process of RIF. CIBERSORT was applied to systematically estimate the infiltration of various immune cells in the endometrium. In addition, the key genes involved in RIF were identified via the protein‐protein interaction (PPI) network and validated in the endometrium from the window of implantation (WOI). Our study provides some new insights into the potential pathogenesis and future research direction of RIF.

## 2 METHODS

### 2.1 Data download and preprocessing

The standardized expression matrix, corresponding microarray platform, and sample information of datasets (GSE26787,^9^ GSE71331,^7^ GSE103465,^14^ and GSE111974^8^) containing RIF and normal controls were downloaded from Gene Expression Omnibus (GEO) database (https://www.ncbi.nlm.nih.gov/geo/). The expression data of RIF and normal controls in each dataset were extracted, and the detailed information of the included datasets was summarized in Table 1. The batch effect was removed using ComBat in the “sva” package (version 3.40.0)^15^ of software R (version 4.1.1), then the four datasets were merged and normalized, and the effect of correction between samples was demonstrated by the principal component analysis cluster diagram. Finally, a combined file containing 14160 genes was used for subsequent analysis.

**TABLE 1:** Information on the datasets including RIF and control samples from GEO

Dataset ID	Platform	Tissue	Sample size (RIF/control)	Biopsy time	Year	Region
GSE26787	GPL570	Endometrium	5/5	7–9 days after ovulation	2011	France
GSE103465	GPL16043	Endometrium	3/3	LH+7	2018	China
GSE71331	GPL19072	Endometrium	7/5	5–7 days after ovulation	2018	China
GSE111974	GPL17077	Endometrium	24/24	LH+7 to LH+10	2019	Turkey

### 2.2 Screening for differentially expressed genes (DEGs)

DEGs between RIF and control endometrial samples were identified using the “limma” package (version 3.48.3),^16^ and the threshold was regarded as *p* < .05 and |log_2_(fold change)| ≥ 1. Then the DEGs were visualized by volcano map using the “ggplot2” package (version 3.3.5).^17^

### 2.3 Functional and pathway enrichment analysis

DEGs were uploaded to Database for Annotation Visualization and Integrated Discovery (DAVID, v6.8)^18^ (https://david.ncifcrf.gov/home.jsp) website for gene ontology (GO) as well as the Kyoto Encyclopedia of Genes and Genomes (KEGG) analysis, additionally *p* < .05 was determined to be statistically significant.

### 2.4 PPI network and identification of hub genes

Statistically significant DEGs were uploaded to the STRING database^19^ (https://string‐db.org/), and the interaction score above .4 between the two DEGs was considered significant. Then the results were visualized by adopting Cytoscape software (version 3.8.2),^20^ and the degree algorithm of the CytoHubba plug‐in was applied to identify hub genes.

### 2.5 Expression of hub genes in the endometrium during the menstrual cycle

The gene expression matrix of GSE4888^21^ and GSE98386^22^ were acquired from the GEO database. Table S1 showed the details of these datasets. The expression levels of hub genes in different menstrual phases were analyzed by “ggplot2” and “ggpubr” (version .4.0) packages. *p* < .05 was considered to be significantly different.

### 2.6 Gene set enrichment analysis

Gene set enrichment analysis (GSEA)^23^ of gene expression matrix was carried out by gseKEGG function in “clusterProfiler” package (version 4.0.5).^24^ The false discovery rate < .25 and *p* < .05 were set as threshold criteria.

### 2.7 Gene set variation analysis

The gene set variation analysis (GSVA)^25^ was performed using the “clusterProfiler” package, and “h.all.v7.5.1.symbols.gmt” was selected as the reference gene set. Then, by applying the “limma” package to analyze the GSVA results, the pathway with noteworthy differences between RIF and the control group can be obtained.

### 2.8 Evaluation and analysis of immune cell infiltration

The proportion of immune cells in the samples from GSE26787, GSE71331, GSE103465, and GSE111974 datasets was estimated using the CIBERSORT method and LM22 signature matrix.^26^ Then “corrplot” package (version .92) and spearman correlation analysis were applied to investigate the interrelationship between 22 kinds of immune cells, and “ggplot2” and “ggpubr” packages were utilized to show the different infiltration of immune cells between RIF and control samples.

## 3 RESULTS

### 3.1 Microarray datasets and identification of DEGs in RIF patients

**FIGURE 1:**
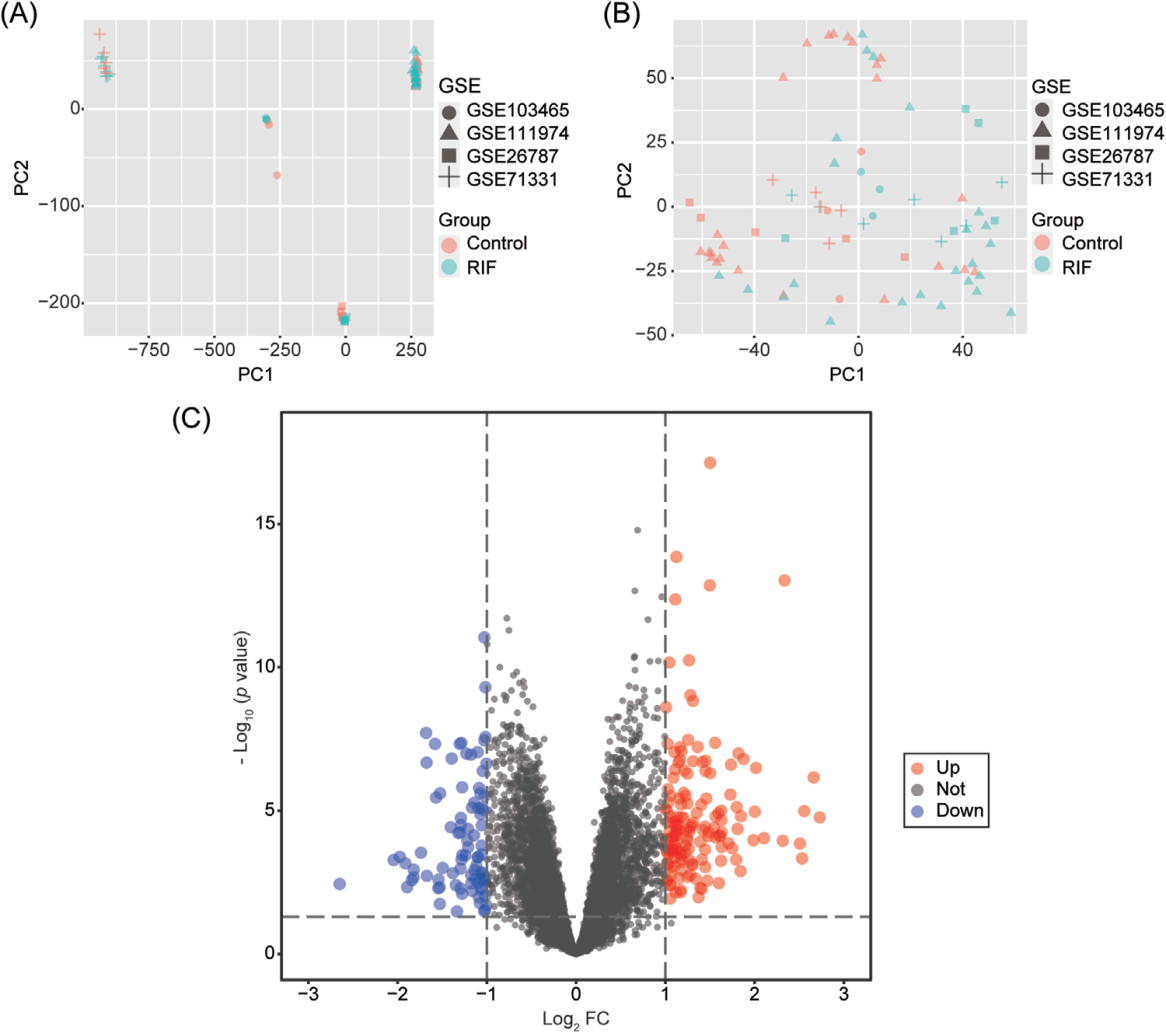
Principal component analysis cluster diagram of samples before and after batch normalization and volcano map of differentially expressed genes (DEGs). Principal component analysis of GSE103485, GSE111974, GSE25787, and GSE71331 datasets before (A) and after (B) normalization. (C) Volcano map of gene expression between RIF and the control group, and there were 151 up‐regulated and 85 down‐regulated genes in the RIF group. FC, fold change

The microarray data of RIF and normal subjects with endometrial samples during WOI were screened and collected from the GEO database. The details of the datasets were shown in Table 1. The inter‐batch difference was removed for further analysis (Figure S1). Principal component analysis results before and after the removal of the batch effect (Figure 1A, B) showed that the clustering of the two groups of samples was more obvious after normalization, indicating that the source of the sample was reliable. The final integrated dataset included 39 RIF patients and 37 healthy controls, with a total of 14160 genes. 236 DEGs were found by the “limma” software package, among which 151 genes were up‐regulated while 85 genes were down‐regulated in the RIF group (Figure 1C and Table S2).

### 3.2 Functional and pathway analysis of DEGs

**FIGURE 2:**
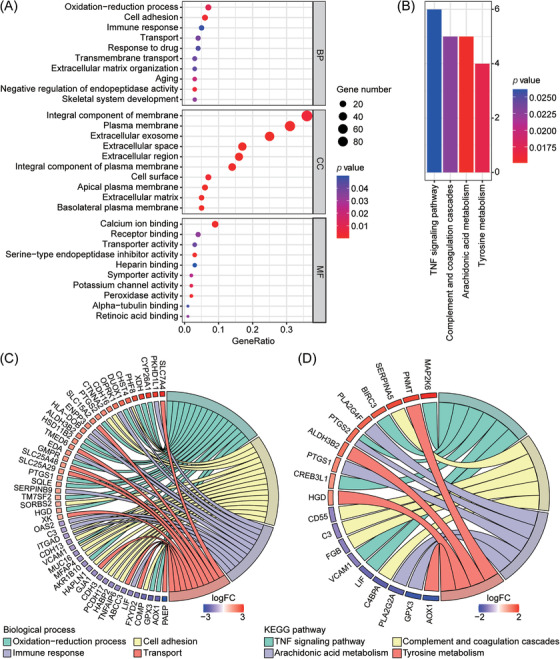
Functional enrichment analysis of DEGs. (A) The top ten GO terms of DEGs, and GO analysis includes three functional groups: biological process (BP), cellular component (CC), and molecular function (MF). (B) KEGG pathway enrichment analysis of DEGs. Chord diagram visualizing the relationship between DEGs and important GO biological processes(C) and the relationship between DEGs and KEGG pathways (D). FC, fold change

The biological function of DEGs was explored by GO and KEGG pathway analysis through the DAVID database. GO enrichment analysis was divided into three functional groups, including biological processes (BP), cellular components (CC), and molecular functions (MF).^27^ As shown in Figure 2A and Table S3, DEGs were significantly enriched in pathways such as oxidation‐reduction process, cell adhesion and immune response in BP category, integral component of membrane, plasma membrane and extracellular exosome in CC category, and calcium ion binding, receptor binding and transporter activity pathways in MF category. Moreover, KEGG pathway analysis showed that DEGs were related to TNF signaling pathway, complement and coagulation cascades, arachidonic acid metabolism, and tyrosine metabolism (Figure 2B and Table S4). At the same time, Chord Diagram was used to further visualize the complex relationships between DEGs and BP of GO terms (Figure 2C) and the KEGG pathway (Figure 2D) to explore the association of various functional annotation categories with RIF‐related genes. These results convey that immune response influences RIF.

### 3.3 GSEA and GSVA

**FIGURE 3:**
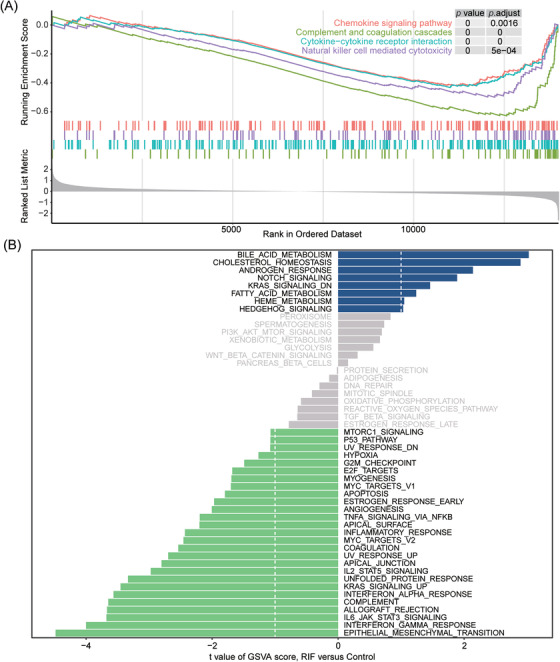
GSEA and GSVA analysis. (A) GSEA of KEGG pathway. (B) GSVA and the differentially expressed analysis of pathways

The GO and KEGG enrichment analysis merely focused on the biological function of the part significantly differential genes, while ignoring the genes with no statistical changes in expression. GSEA detects the expression changes of gene sets rather than individual genes, taking into account the degree of differential expression of all genes, so it can include subtle expression changes. GSEA outcomes revealed that the enriched KEGG pathways were complement and coagulation cascades, cytokine‐cytokine receptor interaction, natural killer cell‐mediated cytotoxicity, and chemokine signaling pathway (Figure 3A and Table S5). Furthermore, GSVA compared the differential expression of 50 classical pathways between RIF patients and normal controls, and the analysis indicated notable differences in the scores of epithelial‐mesenchymal transition, interferon‐gamma response, interferon alpha response, and complement pathways (Figure 3B and Table S6). These findings also propose that immune response has a vital impact on RIF.

### 3.4 Immune cell infiltration

**FIGURE 4:**
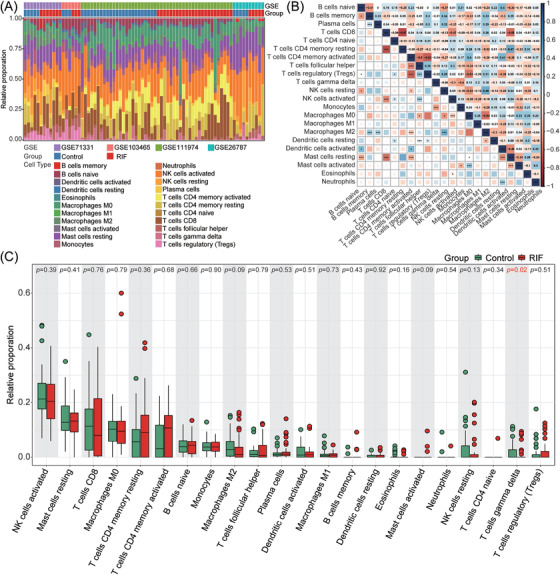
Evaluation and visualization of immune cell infiltration. (A) Composition of 22 types of immune cells in each sample. (B) Correlation of immune cell types in the endometrium from all samples (RIF and normal pregnancy). The intensity of the color and the number in each square indicate the strength of the correlation; blue represents positive correlation, and red represents negative correlation. *p < .05, **p < .01, ***p < .001. (C) The proportion of various immune cell types in the RIF and control group. *p*‐values were obtained using the Mann‐Whitney *U*‐test

The above results illustrate the participation of a variety of immune‐related pathways in RIF. To further investigate the function of immune cell infiltration in RIF, we took advantage of the CIBERSORT method to estimate 22 kinds of immune cells in RIF and normal controls (Figure 4A and Table S7). The correlation map of immune cells showed that NK cells resting was positively correlated with Macrophages M2, Dendritic cells activated was also positively correlated with B cells naïve and T cells CD4 memory activated. While, dendritic cells activated had a remarkable negative correlation with mast cells resting. T cells CD8 was also negatively correlated with T cells CD4 memory resting, NK cells activated and Mast cells resting. T cells CD4 memory activated was negatively correlated with T cells regulatory (Tregs) and T cells follicular helper. Besides, B cells naïve was negatively correlated with B cells memory, and γδ T cells and Monocytes were also negatively correlated (Figure 4B). Afterward, we compared the infiltration of different types of immune cells in RIF and normal controls and found that γδ T cells decreased significantly in the RIF group. While other major immune cell components such as NK cells, macrophages, and CD8 T cells had no difference between the two groups (Figure 4C).

### 3.5 PPI network and hub genes identification

**FIGURE 5:**
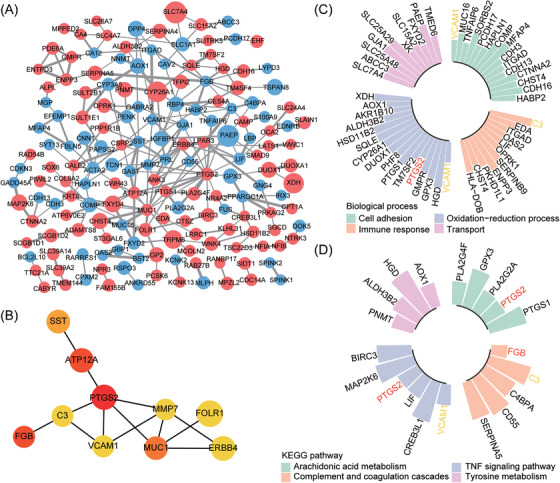
PPI network of DEGs and identification of hub genes. (A) PPI diagram of DEGs. Red nodes represent up‐regulated genes, while blue nodes represent down‐regulated genes, and the thickness of the connecting line represents the interaction score. (B) Ten hub genes. The color of the nodes represents the level of the score ranked by the Degree method using the CytoHubba plugin of Cytoscape, and the redder the color is, the higher the score is. The plot of the hub genes in the GO biological process pathway (C) and KEGG pathway (D), and the height of the column represents the relative mRNA expression of the genes

In order to analyze the interaction of DEGs between RIF and normal endometrium and to figure out the key genes, we uploaded 236 DEGs to the STRING website and then constructed a PPI network via Cytoscape. Subsequently, a PPI network containing 167 nodes and 230 edges was acquired (Figure 5A). Ten genes with the highest connectivity including *PTGS2*, *ATP12A*, *FGB*, *MUC1*, *SST*, *VCAM1*, *MMP7*, *ERBB4*, *FOLR1*, and *C3* (Figure 5B) were selected as hub genes by CytoHubba's degree algorithm. Among them, *PTGS2*, *ATP12A*, *MUC1*, *ERBB4*, and *FOLR1* were up‐regulated in the RIF group, while *FGB*, *SST*, *VCAM1*, *MMP7*, and *C3* were down‐regulated (Figure 5A and Table 2). Meanwhile, to better understand the relationship between hub genes and GO and KEGG enrichment pathways, a schematic diagram of hub genes in the enrichment pathway was constructed. The results came out that the majority of hub genes related to RIF were involved in the regulation of cell adhesion, oxidation‐reduction process, immune response, TNF signaling pathway, and complement and coagulation cascades (Figure 5C, D), indicating an important effect of hub genes on RIF.

**TABLE 2:** Summary of hub genes

Gene Symbol	Description	Fold change
Up‐regulated genes		
PTGS2	Prostaglandin‐endoperoxide synthase 2	2.750
ATP12A	ATPase H+/K+ transporting non‐gastric alpha2 subunit	2.588
MUC1	Mucin 1	2.428
ERBB4	Erb‐b2 receptor tyrosine kinase 4	2.668
FOLR1	Folate receptor alpha	3.019
Down‐regulated genes		
FGB	Fibrinogen beta chain	.474
SST	Somatostatin	.411
VCAM1	Vascular cell adhesion molecule 1	.471
MMP7	Matrix metallopeptidase 7	.493
C3	Complement C3	.489

### 3.6 Hub genes in regulating endometrial receptivity

**FIGURE 6:**
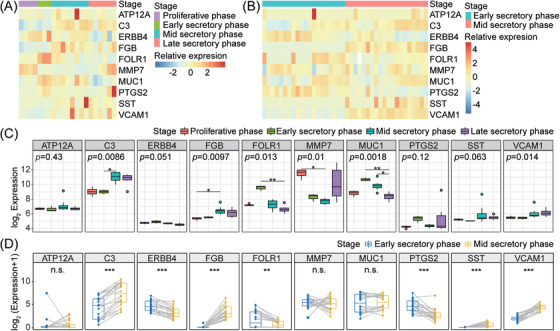
Validation of hub genes in endometrium through the menstrual cycle. Heatmap of hub genes in GSE4888 (A) and GSE98386 (B). Expression of hub genes in different menstrual phases of GSE4888 (C) and GSE98386 (D). Statistical analysis for group comparisons was performed using the Kruskal‐Wallis test (C), and Wilcoxon signed‐rank test (D). n.s. *p* ≥ .05, *p < .05, **p < .01, ***p < .001

In a human menstrual cycle, the endometrium undergoes remodeling, which is driven by substantial gene expression changes.^28^ And only in the mid‐secretory phase, the endometrium will enter a narrow state of receiving embryo implantation, showing a good receptivity. Therefore, the endometrial receptivity in the natural menstrual cycle is space‐time specific. To clarify the relationship between hub genes and endometrial receptivity, we analyzed the expression of hub genes in the normal pregnancy samples including the GSE4888 dataset which contained microarray expression data of four proliferative, three early‐secretory, eight mid‐secretory, and six late‐secretory endometrial samples, and GSE98386 dataset containing the RNA‐sequencing profile form 20 paired early‐ and mid‐secretory endometrial biopsies (Figure 6A, B). Ten hub genes showed dynamic changes during the menstrual cycle (Figure 6C). Compared with the early‐secretory phage, *C3*, *FGB*, *SST*, and *VCAM1* were up‐regulated, while *ERBB4*, *FORL1*, and *PTSG2* were down‐regulated in mid‐secretory endometrium (Figure 6D). It is implied that hub genes have a crucial influence on the establishment of the menstrual cycle and regulation of endometrial receptivity during WOI.

## 4 DISCUSSION

RIF is an important yet unsolved problem in ART, so understanding its possible pathogenesis is helpful to the treatment of this disease and improves the outcome for patients. In this study, we obtained the microarray datasets of RIF endometrium from the GEO database for differential gene expression analysis, and functional enrichment analysis showed an abnormal immune response in RIF patients. Therefore, we further evaluated the infiltration of 22 kinds of immune cells in the endometrium and found that γδ T decreased significantly in RIF patients. At the same time, we found that *PTGS2*, *FGB*, *MUC1*, *SST*, *VCAM1*, *MMP7*, *ERBB4*, *FOLR1*, and *C3* are the key DEGs related to the pathogenesis of RIF. Our study applied a large sample size of endometrial microarray data to identify the DEGs in RIF patients. A variety of bioinformatical analyses were integrated including GO, KEGG pathway enrichment, GSEA, GSVA, and PPI network. Robust DEGs based on the above‐mentioned methods can provide reliable molecular biomarkers for the screening and diagnosis of RIF. We innovatively used the CIBERSORT method to comprehensively evaluate the immune cell infiltration in patients with RIF.

The precise pathogenesis of RIF has not been illuminated yet, and it is generally acknowledged that it is closely related to the immune system, embryo quality, and endometrial receptivity.^29^ Studies have shown that 2/3 of implantation failures are secondary to poor endometrial receptivity.^30^ Even for the best quality blastocysts, their successful implantation depends on the selectivity and acceptability of the endometrium. Therefore, impaired endometrial receptivity has increasingly been considered a potential cause for implantation failure.^31^ By comparing the mRNA expression of the endometrium from RIF and control participants, we found significant abnormalities in the gene expression profile of endometrium in patients with RIF (Figure 1C and Table S2). GO and KEGG enrichment analysis of DEGs indicated that the endometrium of RIF patients had a disordered immune‐related pathway. The immune response is actively involved in the regulation of endometrial receptivity since many pieces of research have illustrated that the expression and functional pathway of immune‐related genes in endometrium and endometrial extracellular vesicles can affect endometrial receptivity and the outcome of embryo transfer.^32^, ^33^, ^34^ In addition, the regulation of the TNF signaling pathway and complement and coagulation cascades may also make the endometrium have the structural and functional changes needed for embryo attachment and growth.^35^, ^36^ Further GSEA and GSVA analysis displayed a significant immune imbalance in the endometrium of RIF patients as well, and the results of GSEA revealed inhibition of complement and coagulation cascades, cytokine‐cytokine receptor interaction, natural killer cell‐mediated cytotoxicity, and chemokine signaling pathway (Figure 3A). Similarly, GSVA analysis showed lower scores of interferon‐gamma response, interferon alpha response, and complement pathway in RIF patients (Figure 3B). Therefore, we can speculate that the abnormal immune status of endometrium in patients with RIF reduces their receptivity to embryos; due to the inability to establish normal maternal‐fetal interaction and immune tolerance, embryos are rejected in the process of multiple transfers, resulting in RIF.

As an indispensable component of the endometrial immune system, immune cells take part in regulating embryo adhesion, trophoblast invasion, vascular remodeling, and immune tolerance.^10^, ^37^ Consequently, it is very important to analyze the infiltration of immune cells in the endometrium. Most previous studies focused on single or selected immune cells in the pathogenesis of RIF, like T cells, NK cells, and so on. But in this study, we took advantage of CIBERSORT to carry out a comprehensive appraisal of immune cell infiltration, including various types of T cells, NK cells, macrophages, DC cells, B cells, and their subsets (Figure 4A and Table S7). It turned out that the decrease of γδ T was correlated with RIF (Figure 4C). γδ T cells are an essential subgroup of “unconventional” T lymphocytes, which bridge innate and acquired immunity and play multiple roles in the maternal immune system.^38^ Compared with non‐pregnant endometrium, the number and percentage of γδ T cells increased significantly during pregnancy.^39^ γδ T cells regulate the differentiation and function of NK cells, macrophages, cytotoxic CD8^+^ T cells, and DC by expressing immunosuppressive molecules such as PD‐1, Tim‐3, CD160 and secreting various anti‐inflammatory cytokines, containing IL‐4, IL‐10, TGF‐β, and IL‐25, and mediate the modulation of T helper‐2 bias in the maternal‐fetal interface to promote the building of immune tolerant microenvironment at the maternal‐fetal interface.^40^ Meanwhile, γδ T cells also have the function of secreting pro‐inflammatory cytokines and cytotoxic molecules, which may exercise their cytotoxic potential in pathological pregnancy.^38^ It has been reported that the percentage of granzyme B^+^ γδ T^41^ and NKG2D^+^ γδ T^42^ cells was significantly higher in peripheral blood lymphocytes of unexplained RIF patients who had failed clinical pregnancy in a subsequent cycle, compared with those who had successful clinical pregnancy. However, the number and function of immune cells in peripheral blood and endometrium are not completely the same or even very different.^43^ Unfortunately, there is a lack of research on the quantity and function of γδ T in the endometrium of patients with RIF. Studies have reported that γδ T cells increase in normal early pregnancy and are preferentially composed of Vδ1 T subsets that play an immunomodulatory role.^44^ In this way, the decrease of γδ T in the endometrium during WOI may damage the establishment of the local immune tolerant microenvironment and ultimately cause the failure of embryo implantation.

Hub genes are usually functionally critical and highly interconnected with other genes in the module. In this study, we identified 10 RIF‐related hub genes: *PTGS2*, *ATP12A*, *FGB*, *MUC1*, *SST*, *VCAM1*, *MMP7*, *ERBB4*, *FOLR1*, and *C3* (Figure 5B). At the same time, the above genes are also involved in the main abnormal pathways in RIF patients (Figure 5C,D), so these genes may play a key role in RIF. What's more, *PTGS2*,^45^ *ATP12A*,^46^ *MUC1*,^47^ *VCAM1*,^33^ *MMP7*,^48^ *ERBB4*,^48^ and *C3* ^35^ identified in our study have previously been demonstrated to participate in regulating endometrial receptivity and embryo implantation. Additionally, our study also found that throughout the natural menstrual cycle, the expression of *PTGS2* and *ERBB4* was significantly down‐regulated during WOI, while the expression of *VCAM1* and *C3* was significantly up‐regulated (Figure 6D), which was opposite to the expression trend of these genes in RIF patients, indicating that the abnormal expression of hub genes impaired the endometrial receptivity and concurrently may forecast the occurrence of RIF. Nonetheless, few studies have reported the effects of *FGB*, *SST*, and *FOLR1* on endometrial receptivity yet. FGB was proposed to not only reduce inflammation by participating in the formation of dense fibrin gels with high antifibrinolytic capacity but also play a key role in regulating endothelial activity and platelet aggregation.^49^ Inflammatory reaction and coagulation processes are assumed to be associated with endometrial receptivity.^50^ SST is significantly upregulated in the hair follicle and contributes to the immune privilege repertoire which is the key to successful skin allograft.^51^ Similarly, the embryo is regarded as an allograft whose implantation and development depend on the proper immune tolerance of the maternal endometrium. FOLR1 performs its biological function by binding folic acid and its reduced derivatives. Previous studies have shown that folic acid may affect the cytotoxicity of NK cells.^52^ It is well known that NK cells play an important part in embryo implantation,^53^ so FOLR1 may regulate endometrial receptivity by affecting NK cells. Unfortunately, we found that SST and FGB were significantly decreased while FOLR1 was up‐regulated in RIF patients, which may damage the endometrial immune tolerance resulting in embryo rejection and consequent implantation failure. To sum up, the specific expression pattern of hub genes during WOI plays an important role in the construction of wonderful endometrial receptivity, and the abnormal expression patterns of these genes will reduce the endometrial receptivity and lead to the occurrence of RIF.

## 5 CONCLUSIONS

In general, our study demonstrated that there were significant abnormalities in the gene expression profile of endometrium in patients with RIF, mainly in the disorder of immune‐related pathways, and found that γδ T cells may be the key immune cells causing RIF. In addition, the identified hub genes potentially play an essential role in the pathogenesis of RIF, which can provide some clues for the prediction and treatment of RIF.

## CONFLICT OF INTEREST

The authors declare no conflicts of interest.

## Supporting information

Supporting information.

Supporting information.

Supporting information.

Supporting information.

Supporting information.

Supporting information.

Supporting information.

Supporting information.

## Data Availability

The data that support the findings of this study are available from the corresponding author upon reasonable request.
