# Supplementary figures and images for: Identification of key genes and immune cell infiltration in recurrent implantation failure: A study based on integrated analysis of multiple microarray studies
Source: Am J Reprod Immunol. 2022 Aug 15;88(4):e13607. doi: 10.1111/aji.13607 (PMC9786880; doi:10.1111/aji.13607)

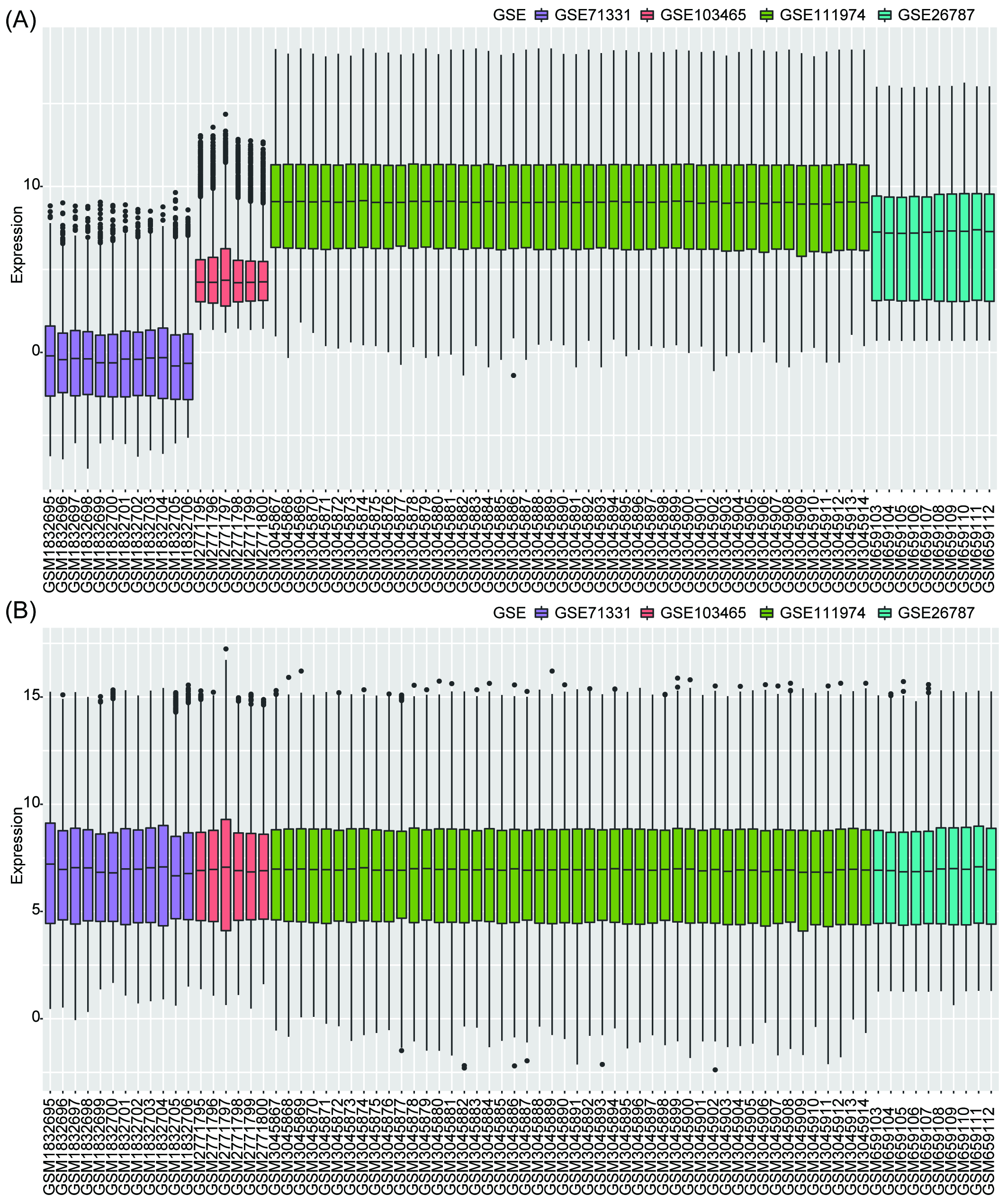

Supplement: Supplementary file 1 — Supporting information. [file AJI-88-e13607-s002.tif]
