# Supplementary material for: Identification of key genes and immune cell infiltration in recurrent implantation failure: A study based on integrated analysis of multiple microarray studies
Source: Am J Reprod Immunol. 2022 Aug 15;88(4):e13607. doi: 10.1111/aji.13607 (PMC9786880; doi:10.1111/aji.13607)
Supplement: Supplementary file 8 — Supporting information. [file AJI-88-e13607-s005.docx]

**FIGURE S1** Integration of GSE103485, GSE111974, GSE25787, and GSE71331 datasets. Boxplots of four datasets before (A) and after (B) removal of batch effect.

| **Dataset ID** | **Platform** | **Tissue** | **Sample size** | **Biopsy time** | **Year** | **Region** |
| --- | --- | --- | --- | --- | --- | --- |
| GSE4888 | GPL570 | Endometrium | 21 | proliferative, early secretory, mid-secretory, and late secretory phase | 2006 | USA |
| GSE98386 | GPL16791 | Endometrium | 40 | early secretory and mid-secretory phase | 2017 | Estonia |

**TABLE S1** Information of the datasets including dynamic endometrial samples
